# Family Resilience, Parenting Styles and Psychosocial Adjustment of Children With Chronic Illness: A Cross-Sectional Study

**DOI:** 10.3389/fpsyt.2021.646421

**Published:** 2021-05-12

**Authors:** Yuan Qiu, Liuqing Xu, Yinzhu Pan, Chunlei He, Yingying Huang, Huan Xu, Zhongqiu Lu, Chaoqun Dong

**Affiliations:** ^1^School of Nursing, Wenzhou Medical University, Wenzhou, China; ^2^Emergency Intensive Care Unit, Emergency Department, The First Affiliated Hospital of Wenzhou Medical University, Wenzhou, China

**Keywords:** family resilience, parenting styles, psychosocial adjustment, mediating effect, children with chronic illness

## Abstract

**Objectives:** To evaluate the level of parent-reported family resilience, parenting styles and psychosocial adjustment of children with chronic illness and to identify the relationships between family resilience, parenting styles and psychosocial adjustment in families with children with chronic illness.

**Methods:** A cross-sectional study was conducted between June 2019 and August 2019. A total of 236 parents of children with chronic illness and 98 parents with healthy children were recruited from general hospitals by convenience sampling. A parent completed the Chinese Family Resilience Assessment Scale, the Parenting Rearing Patterns Questionnaire and the Strengths and Difficulties Questionnaire. Family resilience, parenting styles, and psychosocial adjustment of children with chronic illness were compared with those of healthy children. Structural Equation Modeling (SEM) was performed to explore the mediation effect of parenting styles between family resilience and psychosocial adjustment among children with chronic illness.

**Results:** Parents of children with chronic illness reported lower level of family resilience and authoritative parenting, but more peer relationship problems compared to parents of healthy children. SEM showed that authoritative parenting fully mediated the relationship between family resilience and psychosocial adjustment of children with chronic illness.

**Conclusion:** Childhood chronic illness reduces family resilience, authoritative parenting and children's psychosocial adjustment, but authoritative parenting mediated these effects, so authoritative parenting may be important for family resilience in families of children with chronic illness. Pediatric clinicians and nurses should provide family-centered interventions, as well as parenting training, to improve children's psychosocial outcomes.

## Introduction

It has been reported that 10–20% of children in China suffered from chronic illness, and the prevalence of childhood chronic illness will reach to 29.4‰ by 2020 (1). Chronic illness is a physical or mental condition, which is defined as a process of ‘long duration and generally slow progression that requires ongoing management over a period of years or decades’ (2). Such conditions include diabetes, chronic kidney disease, congenital heart disease, bone/joint disorders, genetic disorders and et al., which are rarely cured completely (2, 3), and are severe challenges to the children and their families (4). In this study, chronic illnesses included endocrine system diseases, neurological developmental diseases, rheumatic system diseases, congenital heart disease, chronic kidney disease and cancers.

The incidence of psychological problems, such as poor social initiative problems, reluctance to participate in group activities, and internalizing problems such as anxiety, depression and social withdrawal was reported to be as high as 21.9% in children and adolescents with chronic illness (5, 6). Meanwhile, childhood chronic illness has also been consistently reported to be correlated with decreased quality of life of family members and impaired family function (7–10).

Despite the above mentioned negative impacts of childhood chronic illness, there is also evidence that some families with children with chronic illness, including mental, emotional, and behavioral disorders (11), pediatric asthma (12), type 1 diabetes (13) and pediatric cancer (14) demonstrate family strength to positively cope with the challenge of chronic illness (15, 16), and take advantage of their stressful experiences to develop stronger relationships, better family cohesion, and positive family belief systems (17, 18).

Walsh (19) defined the family system's capacity to withstand and rebound from adversity, to become stronger and more resourceful as “family resilience.” Family resilience involves there being shared family belief systems, family organizational processes and shared family communication or problems solving processes. Families of children with mental, emotional and behavior disorders can sometimes be more resilient than families in the general population (11). However, family resilience among Chinese families of children with chronic illness remained less studied.

Family resilience may play a significant role in children's psychosocial adjustment, being for instance positively correlated with children's prosocial behavior (20). Family resilience may also mitigate the harmful effects of adverse childhood experience on children's behavioral outcomes (21), while family functioning may be negatively correlated with children's depressive symptoms (22), and positively related to better child adjustment with fewer externalizing problems and less behavioral acting out, as well-greater social competence (14). Family function may be one of the key aspects of family resilience (23), but the relationship between family resilience and child adjustment has not been widely studied. Some studies even propose that family resilience does not necessarily ensure children's psychosocial adjustment, for some children still present with anxiety, depression, learning disabilities and behavior or conduct problems (11). One possible explanation for the differences is the varied measures of family resilience used in previous studies. Therefore, it is necessary to further explore the role of family resilience in the psychosocial adjustment of children with chronic illness.

Here, we chose to use the Chinese Family Resilience Assessment Scale because based on Belsky's (24) process of parenting model. Taraban and Shaw (25) developed an updated process of parenting model, creating broad categories to represent “Family Social Environment,” “Parental Characteristics” and “Child Characteristics,” which supported the interactive associations among family social contextual factors, parents and children. In this model, the influence of the family social environment on child characteristics may be traced back to parenting styles. Therefore, family resilience may interact with family parenting style to affect management of the child's condition, in turn affecting the child's growth and development. Regarding parenting style, Baumrind (26) identified three styles: permissive (few rules or restrictions), authoritarian (harsh and hostile), and authoritative (democratic and warm) (27). Since, a fourth style is generally included; inconsistent parenting, which is sometimes permissive and sometimes harsh and hostile (28).

Parenting style may be affected by stressful life events occurring within families (21), which may result in unhealthy parental emotions that contribute to an increase in inappropriate parenting practices (29–31). In a sample of Latino Youth, authoritative parenting usually occurred within highly cohesive family systems, while authoritarian parenting usually occurred within less cohesive family systems (32). Family resilience may also be related to parental coping and parental emotional support (11). However, the details of the relationship between family resilience and parenting styles remain unexplored. Positive parenting, including authoritative parenting, is predictive of child resilience, self-esteem and life satisfaction (33, 34), while inconsistent parenting may make children aggressive, hostile, opposed, anxious and depressed (35–37). However, the relationship between family resilience, parenting styles and psychological adjustment of children with chronic illness is not well-characterized in contemporary literature.

Understanding the relationships between the psychosocial adjustment of children with chronic illness, family resilience and parenting styles would help to develop interventions to improve the psychosocial well-being of children with chronic illness and their families. This study therefore collected data on parenting style, family resilience and psychosocial adjustment of the child with chronic illness. We hypothesized that (1) families with children of chronic illness would be less resilient, less likely to adopt positive parenting styles, and having children with more psychological and behavioral problems compared to those with healthy children; (2) parenting styles would mediate the relationship between family resilience and the psychosocial adjustment of children with chronic illness.

## Methods

### Participants

A total of 334 parents (236 with children diagnosed of chronic illness, 98 with healthy children) were enrolled in this study. The inclusion criteria for parents of children with chronic illness were: (1) at least having one child aged between 3 and 16 years old with a medically diagnosed uncured chronic illness that had lasted at least 3 months continuously (38); (2) parents aged ≥18 years. Inclusion criteria for the healthy control group were having children never being diagnosed with any chronic health condition. Parents with impaired cognitive function or severe medical conditions as diagnosed by a physician were excluded. Families were also excluded if the target parent and child or any other family member had been diagnosed by a physician as having a history of psychiatric illness or reported currently taking antipsychotic medication. Families including any member with a serious physical or mental illness were also excluded.

### Procedures

This study was approved by the Institutional Ethics Committee of the Medical University (No. 2019017), in accordance with the Declaration of Helsinki. A convenience sampling method was used to collect data between June 2019 and August 2019 from general hospitals in three cities in China (Wenzhou, Ningbo and Shanghai). Parents of children with chronic illness were recruited from the outpatient and inpatient department of the general hospitals. Potential participants were approached by a member of the research team, either in the pediatric outpatient waiting rooms, or in the pediatric inpatient wards of the hospital. In order to ensure that the healthy controls were from similar communities, parents of healthy children were recruited from the medical examination centers of the same hospitals while waiting for their children's annual routine physical examinations. Only one parent per family was interviewed. After screening according to the exclusion criteria and informed consent, all the participants completed a survey questionnaire in a designated room. Data was collected by members of the research team after training from the first author. The entire survey process lasted about 20–30 min, and all participants were provided with a small gift of a plush toy or keychain valued at $2 as compensation. Of the 262 parents of children with chronic illness who agreed to participate, 236 (90.08%) participants returned the complete questionnaire. Of the 108 parents of healthy children who consented to participate, 10 participants were excluded due to incomplete answers, resulting in a valid sample size of 98 (90.74%).

## Measures

### Chinese Family Resilience Assessment Scale

The Family Resilience Assessment Scale, developed by Sixbey (39), was adapted to Chinese culture by Dong et al. (40). The 44-item C-FRAS contains four subscales: family communication and problem solving (27 items); utilizing social and economic resources (eight items); maintaining a positive outlook (six items); ability to make meaning of adversity (three items). Answers to individual items use Likert four-point scales from 1 = strongly disagree to 4 = strongly agree, producing a total score between 44 and 176. Higher scored indicate higher levels of family resilience. Here, Cronbach's α was 0.970, which indicates excellent reliability.

### Parenting Rearing Patterns Questionnaire

Parenting styles were assessed using the Parenting Rearing Patterns Questionnaire (41). It is a 28-item questionnaire with four dimensions: authoritative parenting (10 items); permissive parenting (seven items); authoritarian parenting (three items); and inconsistent parenting (eight items). Answers are scored on five-point scale from 1 (very inconsistent) to 5 (very consistent). The Cronbach's α in the present study was 0.787, which indicates acceptable reliability.

### Strengths and Difficulties Questionnaire

Children's psychosocial adjustment, was assessed by the Strengths and Difficulties Questionnaire (SDQ; (42)). SDQ is a structured questionnaire to screen psychiatric problems of children and adolescents, and parallel versions of the SDQ are available for completion by children's parents, teachers, and children themselves (43). The parent version of SDQ, suitable for children aged 3 to 16 years, was used in this study. It is a 25-item scale with five subscales, including emotional symptoms, conduct problems, hyperactivity/inattention, peer relationship problems and prosocial behavior. All items were scored on a three-point scale 0 to 2 (0, “not true;” 1, “somewhat true;” 2, “certainly true”). The first four subscales (20 items) are summed to generate the total difficulties score ranging from 0 to 40, with higher scores representing a higher degree of difficulties. The last subscale (five items) belongs to the strength questionnaire with total score ranging from 0 to 10. The higher score in the last subscale indicates a higher degree of prosocial behaviors (44). The Cronbach's α in the present study was 0.728, which indicates acceptable reliability.

### Statistical Analysis

Descriptive data were presented as Mean ± SD or frequency (percentage). *T*-tests were conducted to compare the children with chronic illness to the control healthy children. Pearson correlations were calculated to examine the unadjusted correlations between family resilience, parenting styles and psychological adjustment among children with chronic illness, and the SEM (Structural Equation Modeling) was employed to test the mediating effect of parenting styles between family resilience and Children's psychosocial adjustment. The maximum likelihood method was used to construct the model. Chi-square/degrees of freedom (χ2/df), root mean square approximation error (RMSEA), Cumulative Fit Index (CFI), Tucker-Lewis index (TLI) and Incremental Fit Index (IFI) were used to estimate the fit of the model. The model was proved to have a good fit if χ^2^/df < 3, RMSEA < 0.08, SRMR < 0.05, as well as CFI, TLI and IFI > 0.9 (45). Analysis of data was carried out using SPSS Version 25.0 (IBM Corp., Armonk, NY, USA) and Amos 25.0 programs (IBM Corp., Armonk, NY, USA). A *p*-value of < 0.05 was considered significant.

## Results

### Demographic Characteristics

Of the 236 parents of children with chronic illness, 59 were fathers and 177 were mothers, mean age 37.48 ± 6.58 years. Of the 98 parents of healthy children, 24 were fathers and 74 were mothers, mean age 36.78 ± 7.44 years. Children with chronic illness (148 male, 88 female) had a mean age of 8.69 ± 3.62 years, while healthy children (49 male, 49 female) had a mean age of 7.42 ± 3.09 years. Thirty-seven children (15.7%) were diagnosed with endocrine system diseases, including type 1 diabetes mellitus and growth hormone deficits, 12 (5%) with nervous system diseases, including cerebral palsy and multiple sclerosis, 69 (29.2%) with rheumatic system diseases, including juvenile idiopathic arthritis, allergic purpura and systemic lupus erythematosus, 16 (6.8%) with congenital heart disease, 53 (22.5%) with chronic kidney disease, 25 (10.6%) with cancers and 24 (10.2%) with other diseases. The demographic characteristics of the participants and their children were summarized in Table 1. There was no difference between two groups in the parents' age, gender, employment status, children's age and medical insurance. However, the childhood chronic illness group tended to have parents with lower education (χ^2^ = 21.539, *p* = 0.000), families with more children (χ^2^ = 10.187, *p* = 0.001), lower monthly household income (χ^2^ = 15.587, *p* = 0.001) and more male children (χ^2^ = 4.625, *p* = 0.032), see Table 1.

**Table 1:** Demographic characteristic of families of children with chronic illness and families of healthy children.

**Variable**	**Families of children with chronic illness (*n* = 236) Mean ± SD, (*N*) %**	**Families of healthy children (*n* = 98)Mean ± SD, (*N*) %**	***t*/**χ^2^****	***p*-value**
Parent Age	37.48 ± 6.576	36.78 ± 7.441	−0.856^Δ^	0.393
**Parent gender**				
Male	25% (59)	24.5% (24)	0.01	0.922
Female	75% (177)	75.5% (74)		
**Parent occupational status**				
Employed	59.3% (140)	70.4% (69)	3.634	0.057
Unemployed	40.7% (96)	29.6% (29)		
**Parent education level**				
Middle School and below	53.8% (127)	32.7% (32)	21.539	0.000
High School/secondary school	19.5% (46)	14.3% (14)		
College or higher	26.7% (63)	53.0% (52)		
**Monthly household income**				
<3000RMB	17.4% (41)	12.2% (12)	15.587	0.001
3000–5000RMB	28.0% (66)	14.3% (14)		
5001–8000RMB	25.8% (61)	23.5% (23)		
>8000RMB	28.8% (68)	50% (49)		
**The number of children**				
one	29.7% (70)	48% (47)	10.187	0.001
two or more	70.3% (166)	52% (51)		
**Child Age**				
3–6 year	34.3% (81)	45.9% (45)	5.828	0.054
7–12 year	49.6% (117)	45.9% (45)		
13–16 year	16.1% (38)	8.2% (8)		
**Child gender**				
Male	62.7% (148)	50% (49)	4.625	0.032
Female	37.3% (88)	50% (49)		
**Types of chronic illness**				
Endocrine system (Type 1 diabetes mellitus/Growth hormone deficits)	15.7% (37)	–	–	–
Nervous system(Cerebral palsy/Multiple sclerosis)	5% (12)	–		
Rheumatic system (Juvenile idiopathic arthritis/Allergic purpura/Systemic lupus erythematosus/Kawasaki disease)	29.2% (69)	–		
Cardiovascular system (Congenital heart disease)	6.8% (16)	–		
Chronic kidney disease	22.5% (53)	–		
Cancer (Leukemia/Malignancies)	10.6% (25)	–		
Others	10.2% (24)	–		
**Sources of medical expenses**				
Self-paying	44.1% (104)	38.8% (38)	0.794	0.373
Medicare	55.9% (132)	61.2% (60)		

*SD, standard deviation*;

Δ *, t value*.

### Family Resilience, Parenting Styles and Psychosocial Adjustment of Children With Chronic Illness

As shown in Table 2, parents of children with chronic illness scored lower on family resilience and its subscales (*p* < 0.05), and lower on authoritative parenting style (*p* = 0.010) compared to the parents of healthy children. The psychosocial adjustment of children with chronic illness was similar to that of healthy children, except that parents of children with chronic illness reported more their children had more peer relationship problems than parents of healthy children (*p* = 0.029).

**Table 2:** Differences in family resilience, parenting styles and child psychosocial adjustment by childhood chronic illness history (*n* = 334).

	**Children with chronic illness (*n* = 236)**	**Healthy children (*n* = 98)**	***t***	***p*-value**
Family resilience	127.82 ± 9.942	135.27 ± 16.154	4.240	0.000
FCPS	80.66 ± 7.085	84.90 ± 10.305	3.721	0.000
USER	21.44 ± 2.626	22.69 ± 3.565	3.133	0.002
MPA	16.91 ± 2.044	18.43 ± 2.306	5.962	0.000
AMMA	8.81 ± 1.003	9.24 ± 1.347	3.252	0.001
Authoritative parenting	37.44 ± 5.919	39.20 ± 5.183	2.574	0.010
Permissive parenting	22.45 ± 5.471	23.54 ± 5.449	1.662	0.097
Authoritarian parenting	8.83 ± 2.041	8.40 ± 2.389	−1.570	0.118
Inconsistent parenting	23.98 ± 4.941	24.46 ± 6.292	0.668	0.505
Emotional symptoms	2.80 ± 2.021	3.15 ± 2.198	1.430	0.154
Conduct problems	2.16 ± 1.456	2.35 ± 1.386	1.078	0.282
Hyperactivity/inattention	4.39 ± 2.046	4.46 ± 2.188	0.293	0.770
Peer relationship problems	3.36 ± 1.649	2.94 ± 1.545	−2.188	0.029
Prosocial behavior	6.31 ± 2.365	6.38 ± 2.147	0.262	0.794
Total difficulties scores	12.71 ± 4.824	12.90 ± 5.142	0.322	0.748

*FCPS, Family Communication and Problem Solving; USER, Utilizing Social and Economic Resources; MPA, Maintaining a Positive Attitude; AMMA, Ability to Make Meaning of Adversity*.

### Relationship of Family Resilience, Parenting Styles and Psychosocial Adjustment of Children With Chronic Illness

The results of the correlation analysis were shown in Table 3. Family resilience was positively correlated with authoritative parenting (*r* = 0.278, *p* < 0.01) and authoritarian parenting (*r* = 0.190, *p* < 0.01), but negatively correlated with inconsistent parenting (*r* = −0.169, *p* < 0.01). Family resilience was positively related to prosocial behavior (*r* = 0.139, *p* < 0.05) and negatively related to total difficulties (*r* = −0.147, *p* < 0.05). Authoritative parenting was negatively correlated to the total difficulties (*r* = −0.238, *p* < 0.01) and positively correlated to the prosocial behaviors (*r* = 0.326, *p* < 0.01).

**Table 3:** Correlation analysis between family resilience, parenting styles and psychosocial adjustment of children with chronic illness (*n* = 236).

	**1**	**2**	**3**	**4**	**5**	**6**	**7**	**8**	**9**	**10**	**11**	**12**	**13**	**14**
1.FCPS	1													
2.USER	0.370^**^	1												
3.MPO	0.435^**^	0.249^**^	1											
4.AMMA	0.348^**^	0.202^**^	0.377^**^	1										
5.Family resilience	0.935^**^	0.599^**^	0.619^**^	0.480^**^	1									
6.Authoritative	0.317^**^	0.096	0.015	0.237^**^	0.278^**^	1								
7.Permissive	0.038	−0.081	0.003	−0.091	−0.003	0.117	1							
8.Authoritarian	0.194^**^	0.097	0.100	0.059	0.190^**^	0.150^*^	0.223^**^	1						
9.Inconsistency	−0.143^*^	−0.087	−0.156^*^	−0.120	−0.169^**^	−0.054	0.158^*^	0.206^**^	1					
10.Emotional symptoms	−0.060	−0.078	−0.114	−0.095	−0.096	−0.064	0.077	0.032	0.066	1				
11.Conduct problems	−0.038	0.037	−0.022	−0.084	−0.030	−0.239^**^	0.166^**^	0.046	0.086	0.295^**^	1			
12.Hyperactivity/inattention	−0.125	−0.054	−0.090	−0.095	−0.132^*^	−0.211^**^	0.067	−0.081	0.157^*^	0.289^**^	0.478^**^	1		
13.Peer relationship problems	−0.141^*^	0.017	−0.081	−0.081	−0.121	−0.145^*^	0.060	0.034	−0.057	0.283^**^	0.179^**^	0.067	1	
14.Prosocial behavior	0.130^*^	0.095	0.057	0.095	0.139^*^	0.326^**^	−0.170^**^	0.080	−0.065	−0.057	−0.372^**^	−0.368^**^	−0.201^**^	1
15.Difficulties total scores	−0.138^*^	−0.039	−0.120^*^	−0.133^*^	−0.147^*^	−0.238^**^	0.131^*^	−0.004	0.101	0.727^**^	0.689^**^	0.712^**^	0.543^**^	−0.361^**^

*FCPS, Family Communication and Problem Solving; USER, Utilizing Social and Economic Resources; MPA, Maintaining a Positive Attitude; AMMA, Ability to Make Meaning of Adversity*;

* *P < 0.05*,

** *P < 0.01*.

### Mediating Effect of Authoritative Parenting Between Family Resilience and Psychosocial Adjustment Among Children With Chronic Illness

Based on the results of the correlation analysis, it was hypothesized that authoritative parenting would mediate between family resilience and psychosocial adjustment. SEM was employed to test the model, with family resilience as the independent variable, total difficulties and prosocial behavior as dependent variables, and authoritative parenting as the mediating variable. SEM indicated that the measurement model had an acceptable fit to the data, with x^2^/df = 1.565; CFI = 0.941; TLI = 0.913; IFI = 0.944; SRMR = 0.0483, and RMSEA = 0.049.

**Figure 1:**
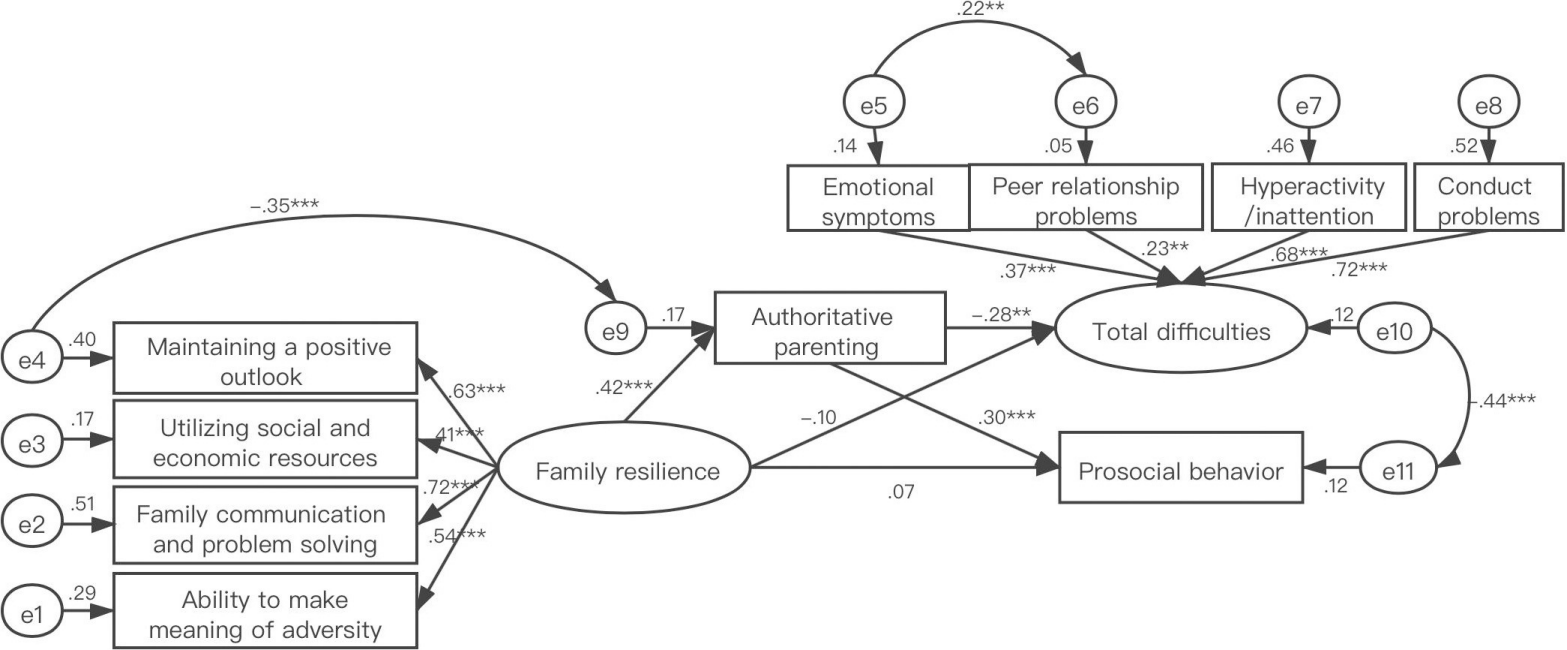
Structural equation model of family resilience, authoritative parenting and psychosocial adjustment. Types of chronic illness as control variable.

The bootstrap bias-corrected estimator was used to further test the above model. As shown in Tables 4, 5 and Figure 1, the standardized path coefficients supported the positive correlation with family resilience and authoritative parenting style (β = 4.552, SE = 1.029, *p* < 0.001), the positive correlation with family resilience and prosocial behavior (β = 0.322, SE = 0.357, *p* = 0.366), and the negative correlation with family resilience and total difficulties (β = −0.133, SE = 0.141, *p* = 0.346). The 95% bias-corrected confidence intervals (CIs) for the direct effect from family resilience to the total difficulties was −0.304 to 0.146, including zero in the 95% CI, indicating that the direct effect was not significant. The 95% CIs for indirect effect from family resilience to the total difficulties via authoritative parenting style was −0.239 to −0.044, and the 95% CIs for the indirect effect from family resilience to prosocial behavior was 0.067–0.216. The 95% CIs did not include zero, indicating the existence of a full mediating effect of authoritative parenting style between family resilience and psychosocial adjustment of children with chronic illness.

**Table 4:** The model path diagram for family resilience, authoritative parenting and psychosocial adjustment of children with chronic illness.

**Variables**	**Unstandardized coefficients**	**Standardized coefficients**	**S.E**.	***T***	***p***
Family resilience → Authoritative parenting	4.552	0.416	1.029	4.424	***
Authoritative parenting → Total difficulties	−0.035	−0.281	0.013	−2.741	0.006
Family resilience → Total difficulties	−0.133	−0.097	0.141	−0.943	0.346
Authoritative parenting → Prosocial behavior	0.119	0.298	0.029	4.251	***
Family resilience → Prosocial behavior	0.322	0.068	0.357	0.904	0.366

*** *P < 0.001*.

**Table 5:** Intermediate, direct, and total effect analysis of authoritative parenting in the family resilience and psychosocial adjustment.

	**Path**	**Effect size**	**Bias-corrected 95%CI**		**Effect proportion (%)**
			**Lower**	**Upper**	
Dependent variable: total difficulties					
	Mediating effect	−0.117	−0.239	−0.044	54.67
	Direct effect	−0.097	−0.304	0.146	45.32
	Total effect	−0.214	−0.398	0.000	
Dependent variable: prosocial behavior					
	Mediating effect	0.124	0.067	0.216	62.63
	Direct effect	0.074	−0.081	0.234	37.37
	Total effect	0.198	−0.052	0.349	

## Discussion

The purpose of this study was to investigate the family resilience, parenting styles and psychosocial adjustment of children with chronic illness and to explore their relationships from the perspectives of parents.

The present study found that parents of children with chronic illness reported lower level of family resilience compared to parents of healthy children. This result suggested that being diagnosed with childhood chronic illness is a stressful event that might weaken the ability of family as a whole to accommodate stressful events together (46–48). Parents of children with chronic illness also reported lower scores on the authoritative parenting compared to their counterparts in present study, which partially support the result of previous study that parents of children with chronic illness tended to be over-involved in children's lives, and their children were more likely to depend on their parents without autonomy (49). Children with chronic illness were reported to have more peer relationship problems compared to healthy children in the present study, which indicates that children with chronic illness might be less capable of dealing with peer relationship problems (50).

As Chinese culture emphasizes interdependence and emotional restraint to maintain harmonious interpersonal relationships (51), our results suggest that authoritarian parenting was positively correlated with family communication and problem solving and total score of family resilience. However, authoritarian parenting did not significantly correlate with any child behavior outcomes in this study. A possible explanation is that children's authoritarian parenting is normal within Chinese culture (52). The permissive parenting style was not significantly correlated with family resilience, but was positively related to total difficulties and weaker prosocial behaviors, which is in consistent with previous research (53–56). Permissive parenting provides minimal structure (57), so may have little influence on how family subsystems interact with one another in terms of their cohesion and flexibility to cope with childhood chronic illness. In the present study, authoritative parenting style was negatively correlated with total difficulties and positively correlated with prosocial behavior among children with chronic illness, as found in previous studies (58, 59), suggesting that authoritative parenting is the most effective parenting style to provide a supportive context for child development (60), possibly providing greater family cohesion and more balanced family functioning (61).

Family resilience was negatively correlated with total difficulties and positively correlated with prosocial behavior, as found previously (62–64). Furthermore, in line with our hypothesis, the present study found that authoritative parenting played a fully mediating role between family resilience and child psychosocial adjustment (both total difficulties and prosocial behavior). This result provides evidence that parenting may be affected by having a child with chronic illness and that the family's perceived ability to cope with the adversity of chronic illness is related to authoritative parenting practices which positively affect the child's emotional and behavioral health (25). Families with high levels of resilience have flexibility in patterns of family organization that enable the family system to hold together in times of crisis, rapidly mobilize crisis management skills, develop new abilities and stronger bonds (65), communicate openly and effectively with their children and maintain close family relationships, which would contribute to positive parenting to affect children's psychosocial adjustment (32).

The mediation analysis helps to clarify that family resilience impacts children's psychological adjustment through authoritative parenting. On basis of these findings, clinicians should conduct family-based surveys and in-depth interviews in future studies to guarantee contact with parents and to obtain valuable information about family variables pertaining to the children's mental health. The focus should be on the link between parenting styles and the children's psychosocial adjustment, providing parents with information about the benefits and recommendations of authoritative parenting styles, and encouraging authoritative parenting as a method of developing family resilience. It is hoped that the development and implementation of such targeted interventions will ultimately improve the psychosocial adjustment of children with chronic illnesses and their families.

### Limitations

This study also has some limitations. First, the cross-sectional design of this study cannot determine the causality relations among the variables. It is necessary for the future studies to employ a longitudinal design to test the mediating models. Additionally, all the questionnaires were filled out by one parent, which may have introduced individual positivity bias across all three questionnaires. Also, the point of view of the children was not investigated. Therefore, the future studies can explore family resilience, parenting styles and psychosocial adjustment focusing on parent-child dyads. Third, there should be with caution about the generalizability of the results of this study since our sample was obtained from general hospitals in three cities of eastern China using convenience sampling and the sample size was modest. Fourth, the severity of the childhood chronic illnesses was not considered in the analysis, which limits the interpretation of the findings.

## Conclusion

Parents of children with chronic illnesses reported that their children had higher levels of psychosocial problems and that the family was less resilient. However, parents who scored higher on authoritative parenting reported more resilience and fewer psychosocial problems for their children. Moreover, authoritative parenting appeared to account for much of the relationship between family resilience and improved child functioning, suggesting that education on parenting style would be helpful for families of children with chronic illness.

## Data Availability Statement

The raw data supporting the conclusions of this article will be made available by the authors, without undue reservation.

## Ethics Statement

The studies involving human participants were reviewed and approved by the Institutional Ethics Committee of Medical University (No. 2019017). The patients/participants provided their written informed consent to participate in this study.

## Author Contributions

YQ: data collection, writing - original draft, writing review, and editing. LX, YP, CH, YH, and HX: data collection, data curation, writing review, and editing. ZL: conceptualization, writing review, and editing. CD: conceptualization, writing - original draft, writing review, and editing. All listed authors meet the authorship criteria and were in agreement with the content of the manuscript.

## Conflict of Interest

The authors declare that the research was conducted in the absence of any commercial or financial relationships that could be construed as a potential conflict of interest.
